# The effect of nutritional education based on the health action process approach (HAPA) on the pregnancy outcomes among malnourished pregnant mothers

**DOI:** 10.1186/s12884-024-06276-7

**Published:** 2024-01-25

**Authors:** Atieh Razzazi, Mark D. Griffiths, Zainab Alimoradi

**Affiliations:** 1 Students research committee, School of Nursing & Midwifery, Qazvin University of Medical Sciences, Qazvin, Iran; 2 International Gaming Research Unit, Psychology Department, Nottingham Trent University, Nottingham, UK; 3 Social Determinants of Health Research Center, Research Institute for Prevention of Non-Communicable Diseases, Qazvin University of Medical Sciences, Qazvin, 34197-59811 Iran

**Keywords:** Health action process approach, Pregnancy outcomes, Nutritional education

## Abstract

**Objective:**

To examine the effectiveness of nutritional education based on the health action process approach (HAPA) on pregnancy outcomes among malnourished pregnant mothers utilizing nutritional support.

**Methods:**

In a randomized controlled trial, 234 malnourished pregnant women under nutritional support from the fourth month of pregnancy participated. Participants were randomly allocated in study groups by the balance block randomization method. Data were collected using a socio-demographic and pregnancy outcomes checklist as well as self-devised questionnaire assessing the constructs of the HAPA model before and three months after the educational intervention. The framework of the educational intervention was based on the constructs of the HAPA and included three one-hour training sessions through lectures, group discussions, ‘question and answer’ sessions, and the use of educational tools. Data were analyzed using analysis of covariance (ANCOVA) and SPSS software.

**Results:**

Pregnancy outcomes including optimal weight gain during pregnancy (*p* = 0.47), neonate’s birth weight (*p* = 0.58), gestational age at delivery (*p* = 0.83), type of delivery (*p* = 0.48) gestational anemia (*p* = 0.22), diabetes (*p* = 0.59) and hypertension (*p* = 0.29) were not significantly different in the intervention and control groups. The results showed that the educational intervention produced a significant increase in the total score (24 points) in the intervention group. Improvement of scores in the intervention group compared to the control was observed in all of the model constructs except outcome expectation (0.68 decrease). The educational intervention in the present study had a large measure of effect in total (SMD: 2.69, partial eta^2^: 0.664).

**Conclusion:**

A nutritional education intervention based on the HAPA model for malnourished pregnant women increased behavioral intention and planning for action to have better nutritional behavior. However, the intervention did not change the pregnancy outcomes significantly.

**Practice implications:**

Nutritional education based on the HAPA model can be used to improve nutritional behaviors of malnourished pregnant women.

## Introduction

Nutrition is a critical part of health and development. Better nutrition is related to improved maternal health (and consequently their children), stronger immune systems, safer pregnancy and childbirth, and lower risk of non-communicable diseases (such as diabetes and cardiovascular disease) during pregnancy [1, 2]. Pregnancy is a critical period during which maternal nutrition and lifestyle choices are major influences on mother and child health [3]. Women’s nutrition, before and during pregnancy, may play a key role in their reproductive health and is recognized as being important for optimizing pregnancy outcomes [4]. Inadequate levels of key nutrients during pregnancy may lead to fetal intrauterine growth restriction, and predisposing the infant to chronic conditions in later life [5]. Improving the well-being of mothers, infants, and children is key to the health of the next generation [3]. Therefore, a healthy diet in pregnancy helps ensure proper fetal growth, good maternal health, and lactation. Consequently, nutritional interventions, education, and counseling need to be an integral part of prenatal care [3, 5].

One of the nutritional interventions is the Special Supplemental Nutrition Program for Women, Infants, and Children (WIC) which provides nutritional support for pregnant and postpartum women and young children. This nutritional support has been shown to improve nutrition among women during this critical period of the life course [6]. Although providing a food basket for a pregnant woman can help maintain a healthy diet throughout pregnancy by creating opportunities for a balanced diet, it is important to note that providing access to food does not mean providing food security for the individual. Because the way food is distributed among family members it may not be based on their needs. Individuals with higher nutritional needs may not be able to eat enough [7]. On the other hand, the nutritional behavior of individuals does not depend only on the availability of access to specific food sources. More specifically, nutritional behavior is a complex set of behaviors that includes the preparation of raw materials, preparation and consumption of foods, eating habits in different cultures, and food policies [8]. Findings from a national study in Australia showed that most Australian women, despite realizing the importance of a healthy diet, did not follow nutritional recommendations [9]. Therefore, one of the important strategies to improve the nutritional status of pregnant women is nutrition education. Nutrition education is an important part of pregnancy that should not be overlooked [10].

Nutrition education is a combination of educational strategies designed to facilitate decision-making in food selection and proper nutritional behaviors that lead to health and well-being [3]. Nutrition education is a practical and important aspect of nutrition that plays an important role in raising public awareness and the health of individuals in society [11]. Most dietary behaviors are related to the knowledge level of the individual, so healthy eating education is important to pregnant mothers [1]. Nutrition knowledge shows that knowledge utility is likely to be related to consumers’ and nutritionists’ particular goals and viewpoints [8]. The majority of pregnant women perceive their diets to be healthy yet they do not consume the recommended daily servings from the five food groups of fruits, vegetables, grains, protein foods, and dairy [9]. Proper planning for designing a beneficial nutritional education includes choosing the right method for nutritional education [12] .

Models of health behavior change postulate a pattern of factors that may improve motivation and eventually lead to sustained behavior change [13]. The health action process approach (HAPA) was developed to help overcome some of the limitation inherent in other models. It is one of the interpersonal models in health education that has been used for nutrition education [13]. The HAPA helps to understand health behavior that always leads to behavior change by providing a solution to the problem and creating a firm intention. Often, the gap between behavioral intent and behavior, referred to in the model as the ‘black box’, is communicated through action planning structures, planning for performance control, or health behavior to facilitate behavior prediction [13, 14].

This model consists of three stages of pre-intention, intention, and action [15]. In the pre-intention stage, the person did not intend to perform the behavior. In the intention stage, the person intended to perform the behavior, but the intention has not yet turned into action. In the action stage, the person performs the desired behavior [13]. This model is different from other social cognitive approaches due to having two distinct phases of action. In this model, the process of changing health behavior includes the motivational phase and the voluntary phase. The motivational phase is the stage in which a person intends to adopt an action or change a risky behavior and in the voluntary phase the person turns their intention into a real behavior and includes three stages: initiation, maintenance, and improvement [15].

The motivational phase focuses on the beliefs that compel a person to perform a particular behavior [16]. Factors such as perceiving risk or paying attention to the situation at risk of health [17], expectations related to the outcome or internal comment about the consequences of an action in a given period [18], and self-efficacy of action or self-confidence to start an action [19] causes the decision-making process leading to behavior. This process eventually leads to the creation of an intention in which individuals prepare themselves to accept the particular behavior and make a decision about it [20]. When a behavioral intention is formed, the person enters the voluntary stage. This phase focuses on the self-regulatory techniques required to plan, initiate, and maintain behavior [21]. This phase includes action planning (creating tangible plans, determining how, when and where the goal will become an action), fulfillment planning (anticipating acceptable barriers, and adopting self-regulatory strategies), self-efficacy resilience (self-confidence in unforeseen challenging situations) and self-efficacy (confidence in resuming behavioral performance after a failure) [13, 19, 21].

Improving the nutrition of pregnant women is not sufficiently effective through food supply interventions only [22]. Moreover, recent studies in the field of lifestyle improvement using the HAPA on healthy individuals in Iranian society (where the present study was carried out) have reported promising results [23, 24]. Therefore, the present study researchers utilized the concepts of the HAPA model as the conceptual framework for designing a nutrition education intervention for pregnant women. More specifically, the present study was designed to investigate the effect of implementing a nutrition education intervention utilizing the HAPA model on the pregnancy outcomes of mothers enrolled on a nutritional support program.

## Methods

### Design

The present study was a randomized controlled trial designed to examine the effectiveness of a nutritional education-based intervention utilizing the health action process approach (HAPA) on pregnancy outcomes among malnourished pregnant mothers receiving nutritional support.

### Participants

All malnourished pregnant women who were enrolled on the nutritional support program from their 16th week of pregnancy were eligible to participate in the study. Mothers who did not visit regularly for prenatal care, and whom were not willing to receive a support basket were not eligible to receive a food basket and subsequently not eligible for the present study.

### Sample size estimation

According to the research done in similar fields [25], with 95% confidence and 95% test power and using G*Power software, as well as an effect size of 0.5 based on meta-analysis [26], the sample size was calculated to be 120 individuals in each of the intervention and control groups.

### Sampling procedure and randomization

First, a list of mothers in the nutritional support program was prepared. Then 240 mothers were randomly selected. These mothers were randomly allocated to study groups. Randomization was carried out using the balanced blocks randomization method with blocks size of four. Using a web random generator, a randomization list was generated. The generated random sequence was then written in papers in the same order from 1 to 240. To conceal allocation sequence, these papers were put in opaque enveloped and numbered with same order from 1 to 240.

### Measures

Data from participants were collected before and three months after the intervention. Three self-devised measures were used. The first part of the survey included demographic questions concerning education, the number of family members of the pregnant mother and spouse, and ownership of the house. The second part of the survey concerned indicators related to pregnancy outcomes including number of pregnancies, number of abortions, gestational age at delivery, body mass index, optimal weight gain during pregnancy, whether the individual had gestational hypertension and gestational diabetes, and the neonatal birth weight.

The third part of the survey included questions assessing the constructs of the HAPA model. This part of the survey comprised 21 questions assessing the six constructs of the HAPA (e.g., intention, risk perception, outcome expectancies, self-efficacy [task, coping, recovery], planning, action control/self-monitoring). Items were designed based on literature review and experts’ opinion to examine HAPA model constructs. Each subscale contained a basic question and a number of related questions. Therefore, after reading the basic question, participants were then asked to rate their responses on a six-point scale from 1 (*absolutely incorrect*) to 6 (*completely correct*). The psychometric properties of measures were assessed using face validity and content validity, and internal reliability was based on the Cronbach’s alpha coefficient in each domain.

#### HAPA construct measurement items

##### Behavioral intention

Three questions were used to assess behavioral intention (e.g., *“I plan to follow a healthy diet next month”*). Internal reliability was very good (α = 0.85).

##### Risk perception

Three questions were used to assess perceived risk (e.g., *“If I do not improve my diet, I will regret it in the future”*). Internal reliability was good (α = 0.75).

##### Outcome expectation

Four questions were used to assess outcome expectation (e.g., *“If I eat healthily daily, the fetus will gain weight”*). Internal reliability was acceptable (α = 0.61).

##### Task self-efficacy

Two questions were used to assess task self-efficacy (e.g., *“Change is always hard. But I’m sure I can improve my nutrition, even if I have to start right away”*). Internal reliability was acceptable (α = 0.65).

##### Coping self-efficacy

Two questions were used to assess coping self-efficacy (e.g., *“I’m sure I can eat healthy foods for a long time to come, even if I’m stressed”*). Internal reliability was acceptable (α = 0.63).

##### Recovery self-efficacy

Two questions were used to assess recovery self-efficacy (e.g., *“I’m sure I can start eating healthily again, even if I’ve been on an unhealthy diet several times”*). Internal reliability was good (α = 0.70).

##### Action planning

Three questions were used to assess action planning (e.g., *“I have carefully planned my diet”*). Internal reliability was good (α = 0.77).

##### Coping planning

Two items were used to assess coping planning (e.g., *“Many times there are obstacles and one has to plan to overcome them”* and *“I have a plan that if I could not eat the food I had in the plan, what other healthy food to eat instead”*). Internal reliability was very good (α = 0.81).

### Intervention procedure and educational content

#### Intervention group

Three monthly educational sessions lasting one hour each were held using combination of interactive education methods including lectures, discussions, and ‘question and answer’ sessions. At the beginning of each session, questions and answers were used to assess people’s awareness of the topics of the session. In the second and third session, the review of the educational content presented in the previous sessions was done to ensure the correct understanding of the concepts by the participants. Education was provided in groups of 9 to 15 individuals. Educational brochures and pamphlets for studying at home and teaching some foods according to the items of the support food basket were planned. All educations were provided by a nutrition specialist. The educational content for each session is provided as below:

#### Session 1

This session aimed to increase the awareness and knowledge, and increase the level of risk perception and expectation of consequences. The education contents were familiarity with the importance of nutrition during pregnancy including introduction to pregnant mothers and explaining the objectives of the research; explaining the importance of nutrition during pregnancy; and describing the serious consequences and important complications associated with unhealthy nutrition.

#### Session 2

This session aimed to increase different aspects of self-efficacy (task, coping and recovery). The education contents were appropriate strategies to overcome the obstacles. This session was designed to help learners and sensitize them to review the perceived barriers to a healthy diet and increase self-efficacy and facilitate overcoming perceived barriers by learning and implementing a healthy diet after commenting on the barriers. Eating a healthy diet and writing about them on a whiteboard were discussed along ways to deal with them. In general, the barriers considered by pregnant mothers in the present study were: lack of sufficient knowledge about the differences in diet during pregnancy, high costs for maintaining a healthy diet, and interest in junk snacks by other family members. These issues were discussed and the nature of each barrier was explained to the pregnant mothers. In order to remove the aforementioned obstacles, in the same training session, with the participation and consensus of pregnant mothers, they worked out solutions to remove the obstacles.

#### Session 3

This session aimed to help participants to plan and to follow a healthy diet. Creating behavioral intent and increasing planning for action and planning for coping were educational objectives. In this session, education regarding emphasis on importance of having dietary planning, how to have such planning, and appropriate solution for continuing operational planning. In order to bridge the gap between intention and behavior in the HAPA model, action planning and performance planning strategies were used. In this session, participants were encouraged to have a healthy diet by providing strategies for having a plan to follow a healthy diet. They were then asked to plan for a healthy diet, especially with regard to items distributed in the support basket. Then the obstacles to planning were identified and solutions to overcome it were offered.

#### Control group

Participants assigned to control group received the routine care and education related to their gestational age, as well as the same food basket as the intervention group.

### Data analysis

In the present study, the collected data were first coded and then analyzed using SPSS software version 24. Central data, dispersion, and proportional statistical tests were used to analyze the data. More specifically, first, using the Kolmogorov-Smirnov test, the data distribution status was examined and confirmed in terms of normality. Comparison between groups was performed to examine the distribution balance of variables based on the proposed Imbens and Rubin method [27] by considering the standardized mean difference less than 0.25 for continuous quantitative variables and risk difference index less than 10% for qualitative variables. Analysis of covariance (ANCOVA) was used to examine the differences between groups in terms of changes in outcome variables by controlling the effect of baseline scores at a significance level of less than 0.05. Three measures of effect including the mean difference (MD), the standardized mean difference (SMD), and the partial eta square based on Cohen’s *d* were calculated to assess intervention effectiveness. Cohen’s *d* 0.2–0.5 is interpreted as “small” effect size, 0.5–0.8 is interpreted as “medium” effect size, and greater than 0.8 is interpreted as “large” effect size. Moreover, partial eta-square is interpreted as: 0.010–0.059: small, 0.060–0.139: medium, and more than 0.140: large [28].

### Ethics

The present study was approved by the regional research committee (approval number: IR.QUMS.REC.1398.209). The study protocol was prospectively registered in the Iranian clinical Trial Registration system under ID of IRCT20180218038789N5 in 24-12-2020. After obtaining the necessary permits, the individuals were invited to participate in the research. Written informed consent was obtained from all participants.

## Results

### Participants characteristics

**Fig. 1:**
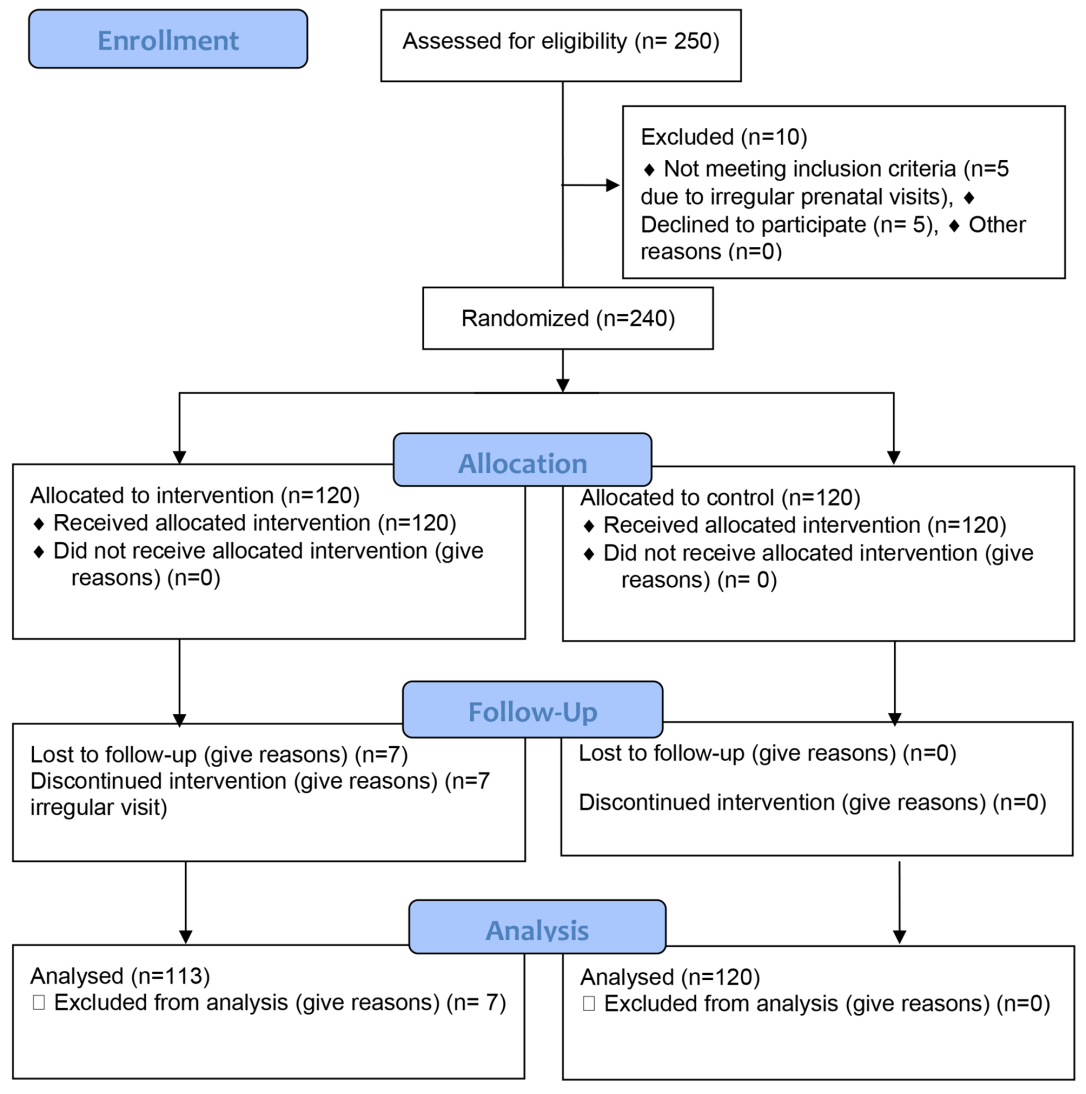
Study flow diagram from recruitment to analysis

The study population comprised 233 pregnant women undergoing a nutritional support program in Qazvin province, Iran. At the beginning of the study, 240 people who met the study criteria were enrolled. These people were randomly assigned to study groups including intervention (120 people) and control (120 people). The questionnaire was completed by all participants at the first visit and before the intervention. In the process of implementing the intervention, all people cooperated fully due to the simultaneity of the educational classes and the distribution of food baskets. In the final stage of completing the questionnaires and after the educational interventions, 120 cases were examined in the intervention group and 113 cases in the control group (7 cases were excluded from the study due to the spread of COVID-19 and lack of access to pregnant mothers). ​Figure 1 presents the study flow diagram from recruitment to follow-up (Fig. 1). The distribution of demographic and reproductive variables in both study groups was balanced (see Table 1).

**Table 1:** Distribution of demographic and obstetrics’ characteristics based on study groups

		Intervention (*N* = 120)	Control (*N* = 120)
		Mean (SD)	Mean (SD)
Age (year)		28.57 (6.73)	29.46 (6.09)
BMI (kg/m^2^)		24.96 (4.66)	24.76 (5.33)
		No (%)	No (%)
Education Stat	Illiterate	11 (9.2)	8 (6.7)
	Elementary	25 (20.8)	29 (24.2)
	High school	28 (23.3)	29 (24.2)
	diploma	27 (22.3)	27 (22.5)
	Academic	29 (24)	27 (22.5)
Job	Housewife	120 (100)	120 (100)
Family size	2	8 (6.7)	7 (5.8)
	3	30 (25)	29 (24.2)
	4	44 (36.7)	43 (35.8)
	5	30 (25)	32 (26.7)
	≥ 6	8 (6.6)	9 (7.5)
Number of children	0	3 (2.5)	6 (6)
	1	34 (28.3)	33 (27.5)
	2	48 (40)	44 (36.7)
	3	29 (24.2)	29 (24.2)
	≥ 4	6 (5)	8 (6.6)
Home ownership	owner	43 (35.8)	41 (34.2)
	rental	77 (64.2)	79 (65.8)
Number of pregnancies	1	21 (17.5)	34 (28.3)
	2	49 (40.8)	40 (33.3)
	3	38 (31.7)	30 (25)
	≥ 4	12 (10)	16 (13.3)
Number of abortions	0	90 (75)	104 (86.7)
	1	25 (21)	13 (10.8)
	≥ 2	5 (4)	3 (2.5)

### Effect of intervention on pregnancy outcomes

Pregnancy outcomes including type of delivery (*p* = 0.48), gestational anemia (*p* = 0.22), gestational diabetes (*p* = 0.59), gestational hypertension (*p* = 0.29), gestational weight gain (*p* = 0.47), neonatal hypothyroidism (*p* = 0.46), baby weight for age (*p* = 0.70), birth weight (grams) (*p* = 0.58) and gestational age at delivery (weeks) (*p* = 0.83) were not significantly different in the intervention and control groups (Table 2). Consequently, the intervention did not affect pregnancy outcomes in intervention group vs. control group.

**Table 2:** Distribution of pregnancy outcome based on study groups

Groups		Intervention (*N* = 120)	Control (*N* = 113)	Results of between group comparison	
Outcome variable		No (%)	No (%)	Statistic	*p*
Type of delivery	Caesarean	72 (60)	63 (55.8)	χ^2^: 0.503	0.48
	Vaginal delivery	48 (40)	50 (44.2)		
Gestational anemia	No	96 (80)	83 (73.5)	χ^2^: 1.48	0.22
	Yes	24 (20)	30 (26.5)		
Gestational diabetes	No	114 (95)	109 (96.5)	χ^2^: 0.288	0.59
	Yes	6 (5)	4 (3.5)		
Gestational hypertension	No	115 (95.9)	111 (98.2)	χ^2^:1.124	0.29
	Yes	5 (4.1)	2 (1.8)		
Gestational weight gain	Normal	69 (57.5)	60 (53.1)	χ^2^: 0.535	0.47
	less than normal	51 (42.5)	53 (46.9)		
Neonatal hypothyroidism	No	116 (96.7)	111 (98.2)	χ^2^: 0.552	0.46
	Yes	4 (3.3)	2 (1.8)		
Baby weight for age	Severely underweight	84 (70)	82 (72.6)	χ^2^: 0.154	0.70
	Underweight	36 (30)	31 (27.4)		
Birth weight (grams)	Mean (SD)	3081.36 (450.79)	3113.81 (442.61)	t (df): -0.555 (231)	0.58
Gestational age at delivery (weeks)	Mean (SD)	38.65 (2.73)	38.59 (1.73)	t (df): -0.214 (231)	0.83

### Effect of intervention on HAPA constructs

The effect of educational intervention on HAPA constructs is shown in Table 3. The results showed that the educational intervention caused a significant increase in the total score of the participants in the intervention group (24 points increase in the total score compared to the control group, *p* < 0.001). Improvement of scores in the intervention group compared to the control group was observed in most of the model constructs except outcome expectation (in this construct, the intervention group’s score decreased in comparison to the control, *p* = 0.05). Considering the two effect size indices (standardized mean difference and partial eta^2^), the educational intervention in the present study had a large measure of effect in total (SMD = 2.96) and in the five subscales of task self-efficacy (SMD = 3.29), action planning (SMD = 2.47), behavioral intention (SMD = 2.27), coping self-efficacy (SMD = 2.14), coping planning (SMD = 1.83), and The effect of intervention was moderate for risk perception (SMD = 0.84), and recovery self-efficacy constructs (SMD = 0.73), and small for outcome expectation (SMD= -0.46). Therefore, the intervention had the greatest effect on task self-efficacy and the least effect on outcome expectation. All changes observed in the constructs and the total HAPA score were statistically significant.

**Table 3:** Results of analysis of variance-covariance (ANOVA-ANCOVA) to investigate the effect of intervention on HAPA subscales

Outcome variable (number of items)	Model*	Time point	Intervention *n* = 121	Control *n* = 113	Mean difference (95% CI)	Cohen’s d (95% CI)	Partial eta^2^	p
Behavioral intention (3 items)		Baseline	12.56 (4.52)	9.44 (5.20)	3.12 (1.86; 4.38)	0.64 (0.38; 0.91)		
	Crude	Post intervention	16.80 (1.35)	9.51 (5.03)	7.29 (6.35; 8.22)	2 (1.69; 2.32)	0.504	< 0.001
	Adjusted	Post intervention	15.95 (2.44)	10.42 (2.44)	5.53 (4.89; 6.18)	2.27 (1.94; 2.60)	0.551	< 0.001
Risk perception (3 items)		Baseline	16.78 (1.83)	12.47 (2.47)	4.31 (3.76;4.86)	1.98 (1.67; 2.29)		
	Crude	Post intervention	17.18 (1.34)	13.08 (2.23)	4.11 (3.64; 4.57)	2.23 (1.91; 2.55)	0.56	< 0.001
	Adjusted	Post intervention	15.60 (1.11)	14.67 (1.11)	0.92 (0.69; 1.25)	0.84 (0.57; 1.10)	0.12	< 0.001
Outcome expectation (4 items)		Baseline	19.96 (3.93)	17.92 (3.08)	2.04 (1.13; 2.94)	0.58 (0.32; 0.84)		
	Crude	Post intervention	19.26 (1.99)	18.83 (2.86)	0.43 (-0.20; 1.06)	0.18 (-0.08; 0.43)	0.008	0.18
	Adjusted	Post intervention	18.73 (1.46)	19.41 (1.47)	-0.68 (-1.07; -0.30)	-0.46 (-0.72; -0.21)	0.05	0.001
Task self-efficacy (2 items)		Baseline	7.88 (2.96)	7.05 (1.66)	0.83 (0.21; 1.45)	0.34 (0.08; 0.60)		
	Crude	Post intervention	11.12 (0.92)	7.28 (1.77)	3.84 (3.48; 4.20)	2.82 (2.46; 3.18)	0.668	0.009
	Adjusted	Post intervention	11 (1.13)	7.42 (1.13)	3.58 (3.28; 3.87)	3.29 (2.90; 3.69)	0.714	< 0.001
Coping self-efficacy (2 items)		Baseline	8.31 (2.88)	6.85 (1.90)	1.46 (0.82; 2.09)	0.60 (0.33; 0.86)		
	Crude	Post intervention	10.55 (1.70)	6.97 (1.87)	3.58 (3.12; 4.04)	2.01 (1.69; 2.32)	0.504	< 0.001
	Adjusted	Post intervention	10.21 (1.34)	7.34 (1.34)	2.86 (2.51; 3.22)	2.14 (1.82; 2.46)	0.526	< 0.001
Recovery self-efficacy (2 items)		Baseline	7.80 (2.95)	5.51 (3.32)	2.29 (1.48; 3.10)	0.73 (0.47; 1)		
	Crude	Post intervention	8.75 (2.93)	5.65 (3.20)	3.11 (2.32; 3.90)	1.01 (0.74; 1.29)	0.206	< 0.001
	Adjusted	Post intervention	7.84 (1.68)	6.62 (1.68)	1.21 (0.78; 1.66)	0.73 (0.46; 0.99)	0.110	< 0.001
Action planning (3 items)		Baseline	11.60 (4.51)	10.30 (4.12)	1.29 (0.18; 2.41)	0.30 (0.04; 0.56)		
	Crude	Post intervention	16.48 (1.08)	10.49 (4.07)	5.99 (5.24; 6.75)	2.04 (1.72; 2.35)	0.513	< 0.001
	Adjusted	Post intervention	16.20 (2.19)	10.79 (2.19)	5.41 (4.84; 5.98)	2.47 (2.13; 2.81)	0.601	< 0.001
Coping planning (2 items)		Baseline	7.81 (3.32)	5.42 (3.26)	2.39 (1.54; 3.23)	0.73 (0.46; 0.99)		
	Crude	Post intervention	11.50 (0.86)	5.74 (3.08)	5.75 (5.18; 6.33)	2.58 (2.23; 2.93)	0.626	< 0.001
	Adjusted	Post intervention	10.98 (2.57)	6.29 (2.57)	4.69 (4.23; 5.15)	1.83 (1.52; 2.13)	0.634	< 0.001
HAPA total score (21 items)		Baseline	92.69 (17.96)	75.11 (17.87)	17.59 (12.97; 22.21)	0.98 (0.71; 1.25)		
	Crude	Post intervention	111.65 (6.24)	77.63 (17.36)	34.03 (30.71; 37.34)	2.66 (2.29; 2.99)	0.638	< 0.001
	Adjusted	Post intervention	106.74 (8.05)	82.89 (8.05)	23.86 (21.66; 29.06)	2.96 (2.59; 3.34)	0.664	< 0.001

The crude model was analyzed using one-way ANOVA, and adjusted models were analyzed using

ANOVA-ANCOVA considering baseline scores as the covariate

## Discussion

The aim of the present study was to determine the effectiveness of a nutritional education-based intervention utilizing the health action process approach (HAPA) on pregnancy outcomes among malnourished pregnant mothers utilizing nutritional support. A total of 233 pregnant mothers participated in the study. The study had two main findings: (i) the pregnancy outcomes were not significantly different the intervention and control groups; (ii) the educational intervention caused a significant increase in the total score of HAPA constructs for the participants in the intervention group compared to the control group with the greatest improvement in task self-efficacy and the least improvement in outcome expectation subscales.

According to the results, the effect of the educational intervention on pregnancy outcomes, including average birth weight, low birth weight, and severe low weight in the intervention and control groups was not significantly different in intervention compared to control group. In a recent systematic review and meta-analysis, evidence regarding effectiveness of counseling and behavioral interventions for healthy weight and weight gain in pregnancy were synthesized and found that counseling and active behavioral interventions to manage gestational weight gain were associated with decreased risk of gestational diabetes, emergency cesarean delivery, macrosomia, and large for gestational age, but not effective in decreasing risk of gestational hypertension, cesarean delivery, or preeclampsia [29]. In a cohort study by Dubois et al. examining the effect of nutrition intervention on pregnancy outcomes among adolescents, the results of multivariate analysis showed that the neonates in the intervention group weighed an average of 55 g more than the neonates in the control group. Their low birth weight and very low birth rate were significantly lower than the non-intervention group [30]. Also, in a study by Gersham et al. [22], nutritional interventions (food packages or fortified food products) were only slightly effective in reducing preterm delivery and had no effect on other pregnancy outcomes [22] that are relevant in relation to the results of the present study. The same study showed that nutrition counseling alone effectively reduced systolic and diastolic blood pressure. They suggested that high-quality clinical trials should be designed and conducted to evaluate the impact of nutritional support interventions along with other methods such as nutritional education and counseling to determine the impact of these interventions on improving pregnancy outcomes [22]. Therefore, it seems that the content and duration of intervention (which was three hours in present study), maternal health status before and during pregnancy, and their access to food supplies are among potential factors which led to different results. The other considerable point regards the lack of significant difference in pregnancy outcomes among both groups might be due to receiving similar nutritional support packages in both groups. In addition, the food items given to the pregnant mother in the support packages were not consumed exclusively by the mother, which was also mentioned and reported in Pinstrup-Andersen’s study on food safety [7]. That study found that providing food packages for a pregnant woman helped them maintain a healthy diet throughout pregnancy by creating opportunities for a balanced diet [7]. However, it is important to note that providing access to food does not mean providing food security for the individual because the way food is distributed among family members may not be based on their needs. Individuals with higher nutritional needs may not be able to eat enough [31]. This can reduce the efficacy of this intervention to a great extent, especially the pregnancy outcomes.

Improvement of scores in the intervention group compared to the control group was observed in total score and all of the HAPA model constructs except outcome expectation. Consistently, a review of relevant literature on application of interventions based on HAPA model constructs for various outcomes including students’ nutritional behavior [32], initiating and continuation of exclusive breastfeeding [33], smoking cessation [34], receiving influenza vaccine [35], oral hygiene among young adults with fixed orthodontic appliance [36], mental health promotion [37], and adherence to treatment in diabetic patients [38] indicates the effectiveness of educational intervention in improving various constructs of the HAPA model.

A decreased score in outcome expectation was not consistent with previous studies. Luszczynska et al. [39] reported increased expectation of outcome after step-by-step intervention on increasing the intention to screen for cervical cancer. Also, Payaprom et al. [35] reported an increased expectation of outcome in educational intervention based on the HAPA model to increase influenza vaccination among high-risk individuals. This was not consistent with the results of the present study. This inconsistency might be due to definition of expectation and participants’ point of view regarding their own ability to reach such outcome. One of example of scale item was *“If I follow a healthy diet, I will not get gestational diabetes”.* Outcome expectation is a person’s beliefs about the desired results if they change their behaviors. It seems that the participants in the present study believed that they could not perform as they were instructed and their expectation of consequences in them decreased. A person’s plan to perform a particular behavior depends on anticipating the consequences and benefits that will result from that behavior. The anticipated benefits of action are psychological visualizations of positive or reinforcing consequences of behavior [40]. Individuals tend to spend their time and resources on activities that are more likely to have positive consequences. The benefits of performing a behavior may be internal or external [13]. Therefore, it is suggested that among vulnerable groups, such as malnourished pregnant women, more attention be paid to the expected outcome structure in the training program. The benefit of using the structure is the expectation of consequences in the planning and implementation of educational interventions, and the impact of the structure on other structures. In educational interventions, by providing opportunities for interaction and action between the learner and the educator, the grounds for eliminating self-control and accepting the views of others are provided.

### Strengths and limitations

To the best of the authors’ knowledge, the present study is the first that has used a randomized controlled trial with a concurrent control group to investigate the effect of educational intervention on the health process pattern on the pregnancy outcomes of malnourished pregnant women enrolled on a nutritional support program. The use of a randomized controlled trial design, simultaneous control group, appropriate sample size, and the use of an educational model in educating vulnerable pregnant women are among the strengths of the present study. However, in interpreting the findings of the present study, it is necessary to pay attention to the following limitations. One of the limitations was the low economic status of all participants and the fact that they all had access to a family support food basket with single items. Although the training sessions tried to identify barriers and teach appropriate strategies to improve nutrition, sometimes (despite the insufficiency of food items in the support basket) individuals could not afford to buy more needed items. In addition, food was often given priority among family members (e.g., children, husbands) and pregnant women did not receive enough food despite their greater need. In addition, conducting the present study at the same time as the first wave of the COVID-19 pandemic increased the economic problems of the participants’ families.

## Conclusion

The results of the present study showed that nutrition-based educational intervention based on the constructs of the HAPA model can improve behavioral intention, planning for action, and self-efficacy among participants, which can lead to performing the appropriate behavior. However, considering that this educational intervention had no significant effect on pregnancy outcomes, it seems that the provision of the educational intervention at the time of preconception care (when couples are planning for pregnancy) or at the beginning of pregnancy, as well as evaluating the economic status and access of mothers to food, the possibility of choosing the right food and ensuring their adequate food intake during pregnancy should be considered as a part of the educational intervention.

### Practice implications

Nutritional education based on the HAPA model can be used to improve nutritional behaviors of malnourished pregnant women.

## Data Availability

The datasets used and/or analysed during the study are available from the corresponding author upon reasonable request.
