# Correction: The effect of nutritional education based on the health action process approach (HAPA) on the pregnancy outcomes among malnourished pregnant mothers

**DOI:** 10.1186/s12884-024-06608-7

**Published:** 2024-06-10

**Authors:** Atieh Razzazi, Mark D. Griffiths, Zainab Alimoradi

**Affiliations:** 1 Students research committee, School of Nursing & Midwifery, Qazvin University of Medical Sciences, Qazvin, Iran; 2 International Gaming Research Unit, Psychology Department, Nottingham Trent University, Nottingham, UK; 3 Social Determinants of Health Research Center, Research Institute for Prevention of Non- Communicable Diseases, Qazvin University of Medical Sciences, Qazvin, 34197-59811 Iran

**Correction: BMC Pregnancy Childbirth 24, 83 (2024)**

10.1186/s12884-024-06276-7.

Following publication of the original article [1], the authors reported an error in the references and in Fig. 1.

First, the name and author year of one of studies used in discussion section is not correct.

- The incorrect citation is: Gersham et al. (2014)
- The correct citation is: Gersham et al. (2016) which refers to reference [22] in the list.

Second, reference [22] has the same details with reference [31]. Reference [31] should be removed and renumber all affected references.

Third, the word irregular in Fig. 1 is not correctly written.

Below is the incorrect Fig. 1.

**Fig. 1:**
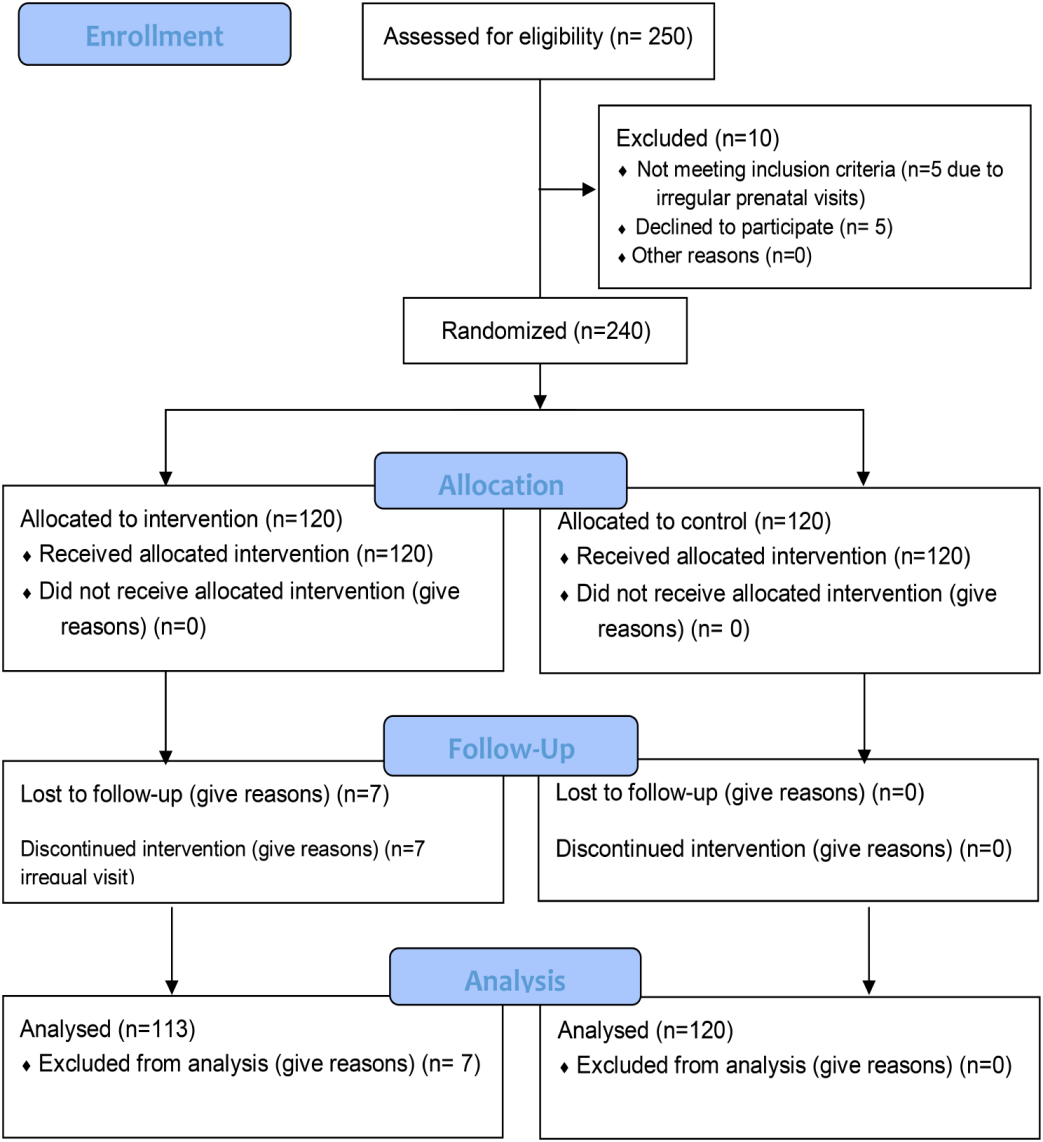
Study flow diagram from recruitment to analysis

Below is the correct Fig. 1.

**Fig. 1:**
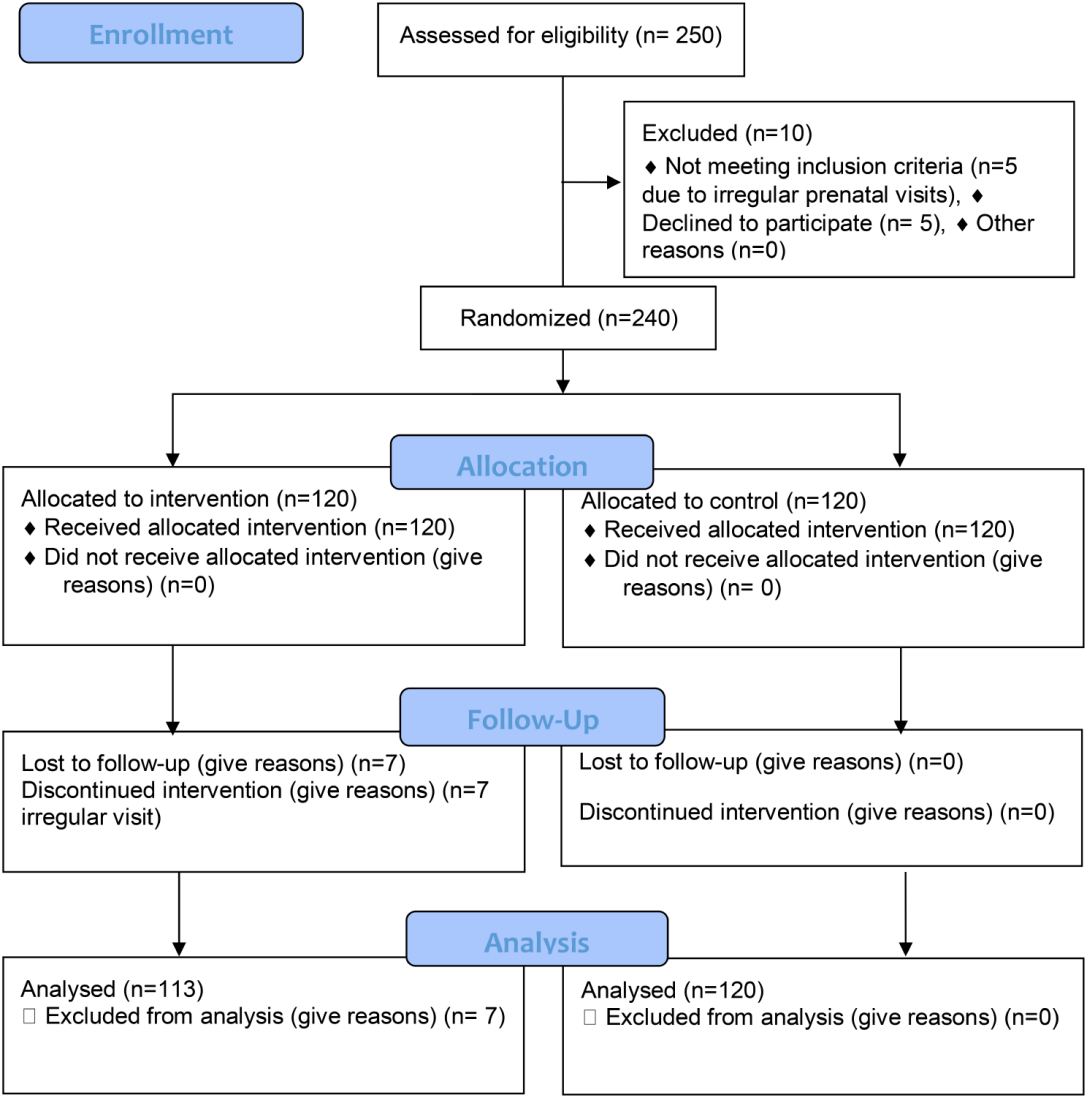
Study flow diagram from recruitment to analysis

The original article has been corrected.
